# Therapeutic effect of two transition metal coordination polymers on ovarian cancer by regulating the expression of estrogen receptor

**DOI:** 10.1080/15685551.2022.2033432

**Published:** 2022-02-09

**Authors:** Jin Zhao, Lan Du

**Affiliations:** a Department of Gynaecology, The Second Affiliated Hospital of Xi’an Medical University, Xi’an, Shaanxi, China; b Department of Gynaecology, Xi’an Angel Women’s and Children’s Hospital, Xi’an, Shaanxi, China

**Keywords:** Coordination polymer, ovarian cancer, CCK-8 assay

## Abstract

In the present study, via using a ligand featuring oxalamide groups N,N′-bis(4-phthalic acid) (H_4_L), two new Cu(II) and Co(II)-containing coordination polymers with the chemical formulae of [Cu_2_L(H_2_O)_4_]_n_ (**1**) and [Co(H_2_L)(H_2_O)_2_]_n_ (**2**) have been successfully prepared via reaction of the corresponding metal salts with the H_4_L ligand. The as-prepared two coordination polymers have been studied via the single crystal X-ray diffraction, elemental analysis, powder X-ray diffraction and thermogravimetric analysis. Their therapeutic effect and mechanism for ovarian cancer was evaluated and explored. Firstly, the inhibitory activity of the new compounds on the proliferation of the ovarian cancer was measured with CCK-8 assay after compound treatment. Besides, the relative expression of the estrogen receptor on the ovarian cancer cells after compound treatment was also determined with real-time RT-PCR assay.

## Introduction

Ovarian cancer is the most well-known gynecological malignancy. Estrogen receptor and estrogen play an important role in the proliferation and apoptosis of ovarian cancer cells [1]. Estrogen receptors have a good predictive role in the prognosis and treatment of breast cancer. Although related studies have been carried out in ovarian cancer in recent years, its role in tumors has not been clarified [2,3]. Estrogen receptor may become a potential post-curing biomarker for ovarian cancer and the key to multi-site combined targeted therapy.

Coordination polymers (CPs) composed of metal ions/clusters and organic ligands applied for the treatment of cancer have attracted many biological and chemists in recent years [4–6]. Because of some structural and physicochemical fractures, CPs can exhibit an interesting therapeutic outcome with a potential synergic effect of its various components. An assembly of biologically active metal ions and biorelevant organic pillars (metabolic intermediates) into metal–organic network provides an appealing way for designing new bioactive materials [7–10]. Among various metal centers, Cu(II) and Co(II)-based coordination polymers are widely studied for their potential anticancer activity values [11–13]. For instance, Wang and co-workers have discovered that the Cu-based polymer particles may act as novel metal-based anticancer drugs in the future because of their potent in vitro anticancer activities against three chosen cancer lines MCF-7, HeLa, and NCI-H446 [12]; Raja et al. synthesized a cobalt(II) coordination polymer of isonicotinic hydrazine exhibiting significant activity against HeLa, HEp-2 and A431 cell lines, which is almost equal to the activity of the well-known anticancer drug cisplatin [14]. Recently, Zheng and co-workers reported the anticancer activity of two Co(II)-based coordination polymers against the glioma cells [15]. In the present study, via using a ligand featuring oxalamide groups N,N′-bis(4-phthalic acid) (H_4_L), two new Cu(II) and Co(II)-containing coordination polymers with the chemical formulae of [Cu_2_L(H_2_O)_4_]_n_ (**1**) and [Co(H_2_L)(H_2_O)_2_]_n_ (**2**) have been successfully prepared via reaction of the corresponding metal salts with the H_4_L ligand. The as-prepared two coordination polymers have been studied via the single crystal X-ray diffraction, elemental analysis, powder X-ray diffraction and thermogravimetric analysis. The structural analysis results show that complex **1** features a 2D grid-like layer while complex **2** demonstrates a 4-connected 3D PtS framework based on 1D [Co(CO_2_)_2_]_n_ chains. After serial biological experiments, the therapeutic effect and mechanism of the synthetic compounds for ovarian cancer was evaluated and explored.

## Experimental

### Chemicals and measurements

All chemical reagents are commercially available without further purification. The IR spectra were taken using the Bruker AXS TENSOR-27 FT-IP spectrometer (Bruker, Karlsruhe, Germany) with pressed KBr pellets in the range of 400–4000 cm^−1^ at room temperature. Elemental analyses of C, H and N were conducted on a PE 240C automatic analyzer (Perkin-Elmer, Waltham, USA) at the analysis center of Liaoning Normal University. The XRD experiments were carried out with a Rigaku D/max2500v/pc X-ray diffractometer (Cu K α radiation, λ = 1.5406 Å). The range of 2θ values was from 0° to 60°, and the scanning rate was 5° /min. The operating voltage and current were maintained at 40 kV and 150 mA, respectively. The simulated XRD powder patterns based on single crystal data were prepared using the Mercury software [16]. Thermogravimetric analysis (TG) was performed on a PerkinElmer Diamond TG/DTA under atmosphere from room temperature to 800°C with a heating rate of 10°C/min.

### Preparation and characterization for [Cu_2_L(H_2_O)_4_]_n_ (1) and [Co(H_2_L)(H_2_O)_2_]_n_ (2)

A mixture of Cu(NO_3_)_2_ · 3H_2_O (24 mg, 0.1 mmol), H_4_L (20.8 mg, 0.05 mmol) and 7.5 mL of distilled H_2_O/MeCN (1:2 v/v) were sealed in a 25 mL Teflon reactor and heated at 105°C for 3 days. After cooling to room temperature at a rate of 10 °C·h^−1^, blue block crystals of **1** were obtained. The obtaining product presented blue massive crystals that were cleaned through EtOH to produce pure samples that are directly used in the single crystal X-ray diffraction studies and powder X-ray diffraction measurements. Yield: 17.1 mg (59.2%, based on H_4_L). Anal. calcd for C_9_H_8_NO_7_Cu: C, 35.36; H, 2.64; N, 4.58. Found: C, 35.29; H, 2.51; N, 4.78%.

The red yellow prism crystals of **two** were obtained by the identical procedure to **1** except that Co(NO_3_)_2_ · 6H_2_O (30 mg, 0.1 mmol) was used instead of Cu(NO_3_)_2_ · 3H_2_O. The obtaining product presented pink massive crystals that were cleaned through EtOH to produce pure samples that are directly used in the single crystal X-ray diffraction studies and powder X-ray diffraction measurements. Yield: 16.8 mg (63.2%, based on H_4_L). Anal. calcd for C_18_H_14_N_2_O_12_Co: C, 42.45; H, 2.77; N, 5.50. Found: C, 4254; H, 2.61; N, 5.45%.

The X-ray data were obtained by utilizing the SuperNova diffractometer. The intensity data was analyzed by utilizing the CrysAlisPro software and converted to the HKL files. The SHELXS program on the basis of direct approach was utilized to create the initial structural models, and the SHELXL-2014 program on the basis of the least-squares approach was modified. The whole non-H atoms were mixed with anisotropic parameters. Then, we utilized the AFIX commands to fix the whole H atoms geometrically on the C atoms that they attached. Table 1 details refinement details as well as crystallographic parameters of the two complexes.

**Table 1.** Refinement details and crystallographic parameters for complexes **1** and **2.**

Identification code	1	2
Empirical formula	C_9_H_8_CuNO_7_	C_18_H_14_CoN_2_O_12_
Formula weight	305.70	509.24
Temperature/K	296.15	296.15
Crystal system	triclinic	monoclinic
Space group	P-1	C2/c
a/Å	5.1362(11)	31.012(3)
b/Å	8.5598(17)	8.3680(10)
c/Å	11.972(2)	7.3950(15)
α/°	104.0690(10)	90
β/°	95.824(7)	101.725(2)
γ/°	106.225(2)	90
Volume/Å^3^	482.07(16)	1879.0(5)
Z	2	4
ρ_calc_g/cm^3^	2.106	1.800
μ/mm^−1^	2.297	0.991
Data/restraints/parameters	1673/0/163	1741/6/172
Goodness-of-fit on F^2^	1.064	1.185
Final R indexes [I ≥ 2σ (I)]	R_1_ = 0.0408,ωR_2_ = 0.1011	R_1_ = 0.1096,ωR_2_ = 0.2379
Final R indexes [all data]	R_1_ = 0.0499,ωR_2_ = 0.1076	R_1_ = 0.1190,ωR_2_ = 0.2420
Largest diff. peak/hole/e Å^−3^	0.46/-0.62	0.61/-0.67

### Cell Counting Kit-8 assay

The Cell Counting Kit-8 assay was conducted in this present research to determine the inhibitory activity of compounds **1** and **2** on the ovarian cancer cells proliferation. This preformation was carried out strictly in accordance with the instructions with only a little modification. In brief, the ovarian cancer cells in the logical growth phage were collected and seeded into the 96 well plates at the destiny of 5000 cells per well, then the plates were placed in an incubator at the condition of 37°C, 5%CO_2_ for 12 hours. After that, compounds **1** and **2** were added into the wells for treatment at serial different concentrations. Then, the culture medium was discarded and fresh medium containing 10 μL CCK-8 reagent was added for further incubation. Finally, the absorbance of each well was determined at the 450 mm. This experiment was conducted at least three times, and the results were presented as mean ± SD.

### Real time RT-PCR

To determine the relative expression levels of estrogen receptor on the ovarian cancer cells after compound treatment, the real-time RT-PCR was conducted in this study totally according to the manufactures’ protocols with some modifications. In short, the ovarian cancer cells in the logical growth phage were collected and seeded into the 6 well plates, then the plates were placed in an incubator at the condition of 37°C, 5% CO_2_ for 12 hours. After that, the compound was added into the wells for treatment at indicated concentrations. Next, the cells were harvested and the total RNA in the cells was extracted with TRIZOL reagent. After measuring the quality and quantity of the total RNA with OD260/OD280, which was then reverse transcript into cDNA. Finally, the relative expression of the estrogen receptor on the ovarian cancer cells after compound treatment was measured with real time RT-PCR, with *gapdh* as the internal control. This experiment was conducted at least three times, and the results were presented as mean ± SD.

## Results and discussion

### Crystal structures

**Figure 1.**
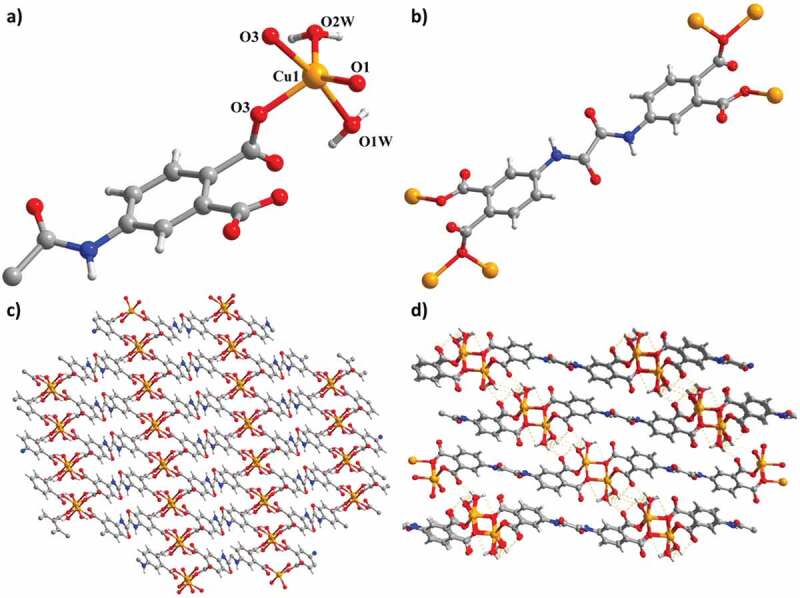
(a) The complex **1**’s asymmetry unit. (b) The coordination pattern for the ligand. (c) The 2D layered network of **1**. (d) The H-bond interactions between the adjacent layers.

The structural solution and refinements results based on the single crystal data collected around room temperature reveal that complex **1** reveals a 2D grid-like layer and belongs to the triclinic space group *P*ī. The asymmetric unit of **1** is composed of one crystallographically independent Cu^2+^ ion, half a fully deprotonated L^4-^ ligand and two coordinated water molecules (Figure 1(a)). Cu1 is five-coordinated by two water O atoms and three carboxylate O atoms from three L^4-^, lying in a distorted trigonal bipyramid geometry (Cu-O = 1.964(3)–2.203(3) Å). Four carboxylate groups adopt different *μ*_1_-*η*^1^,*η*^1^ and *μ*_2_-*η*^2^,*η*^0^ coordination modes (Figure 1(b)). Two Cu^2+^ ions are bridged by two carboxylate single O atoms from two L^4-^ to form a centrosymmetric binuclear parallelogram Cu_2_O_2_ core. Adjacent cores are linked by four carboxylate groups from four L^4-^ to give rise to a 2D grid-like layer (Figure 1(c)). Since both Cu_2_O_2_ cores and L^4-^ are 4-connected nodes, **1** forms a (4,4)-connected grid-like network. Meanwhile, two coordinated H_2_O molecules as H-donors form a pair of interlayer hydrogen bonds with uncoordinated carboxylate O atoms of L^4-^ from adjoining layers (O(1 W)···O(2) =  2.78 Å, O(2 W)···O(2) =  2.70 Å), producing a 3D supramolecular framework (Figure 1(d)).

**Figure 2.**
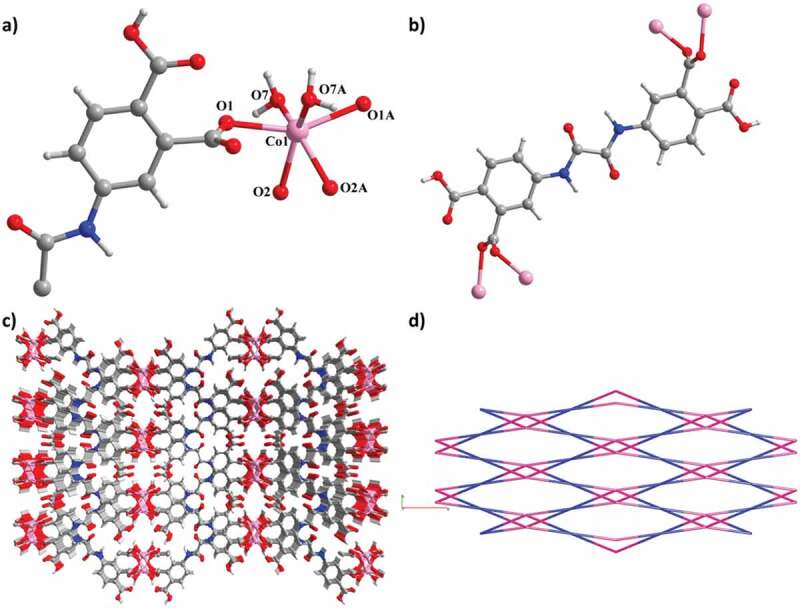
(a) The asymmetry unit view of **2**. (b) The coordination pattern of the ligand. (c) The 3D framework of **2**. (c) The PtS net for **2.**

The single crystal X-ray diffraction study shows that complex **2** shows a 3D framework structure belonging to monoclinic space group *C*2/c. The asymmetric unit of **2** is composed of half a Co^2+^ ion, half a partially deprotonated HL^2-^ ligand and one H_2_O ligand (Figure 2(a)). The Co^2+^ ion (Co1) that is located in the two-fold axis is coordinated by four carboxylate group O atoms of four HL^2-^ and two H_2_O molecules, forming an inerratic trigonal anti-prismatic geometry instead of the common octahedron (Co-O = 2.091(1)–2.274(7) Å). In H_2_L^2-^, two carboxylate groups are coordinated with the same *μ*_2_-*η*^1^,*η*^1^ bridging mode, while the other two are uncoordinated (Figure 1(b)). Two Co^2+^ ions in **2** are connected by a pair of carboxylate bridges of two HL^2-^ to produce an 8-membered (Co_2_C_2_O_4_) subring (Figure 2(b)). Adjacent subrings are interconnected along the *c* axis to generate a 1D [Co(CO_2_)_2_]_n_ chain, which is then extended by HL^2-^ to afford a 2D layer. Moreover, owing to the existence of other HL^2-^ that is almost perpendicular to the layer, a final 3D framework is built (Figure 2(c)). Topologically, each Co^2+^ ion can be simplified as a distorted tetrahedral node, while HL^2-^ is viewed as a square planar node, so **2** forms a 4-connected PtS topology by Topos calculation, which is composed of the equal ratio of tetrahedral and planar nodes having the same point symbol of (4^2^ · 8^4^) (Figure 2(d)).

**Figure 3.**
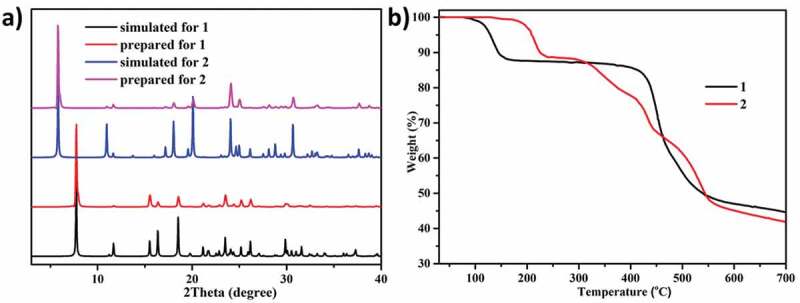
(a) The PXRD patterns for **1** and **2**. (b) The TGA curves for complexes **1** and **2.**

To check the phase purity of the products, powder X-ray diffraction (PXRD) experiments have been carried out for these complexes (Figure 3(a)). The peak positions of the experimental and simulated PXRD patterns are in good agreement with each other, indicating that the crystal structures are truly representative of the bulk crystal products. The differences in intensity may be owing to the preferred orientation of the crystal samples. TGA was performed to estimate the thermal stability of **1**–**2** under N_2_ atmosphere (Figure 3(b)). The weight loss processes in **1** shows two steps, and the first weight loss of 11.9% between 80 and 150°C was caused by the release of two coordinated H_2_O molecules (calcd. 11.9% in **1**). After a thermal stability flat in 150–380°C, the backbone is decomposed. Complex **2** exhibits almost coincident weight loss process, in which the H_2_O molecules were released below 220°C with the weight losses of 10.2% for **2** (calcd 6.9%). After a narrow thermal stability flat, **2** begins to decompose.

### Compound significantly reduce the ovarian cancer cells proliferation

**Figure 4.**
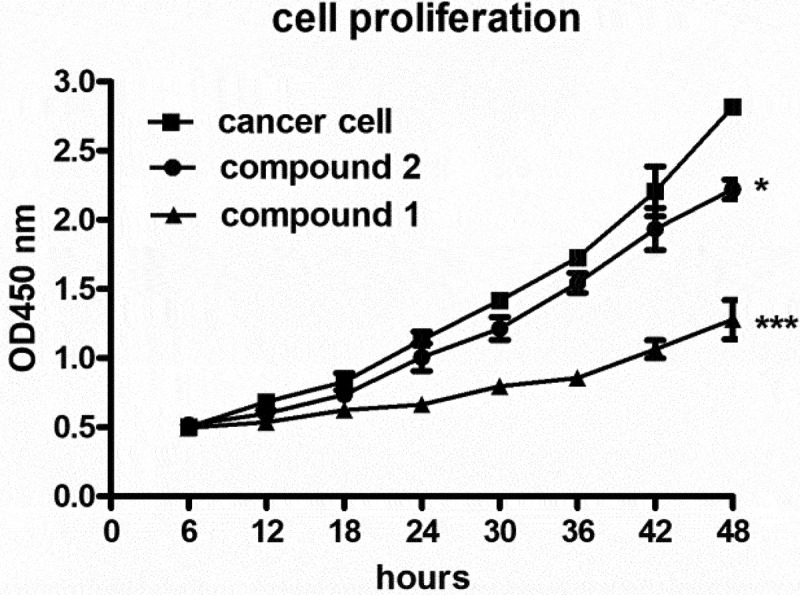
Significantly reduced proliferation of the ovarian cancer cells after compounds treatment. The ovarian cancer cells in the logical growth phage were collected and seeded into the 96 well plates, compound **1** or **2** was added for treatment at serial different concentrations for 48 hours treatment. The proliferation of the ovarian cancer cells was determined with CCK-8 assay. * means P < 0.05, *** means P < 0.005.

After the synthesis of compounds **1** and **2** with novel strucutres, their biological activity against ovarian cancer was determined. Thus, the CCK-8 detection was firstly carried out in this research to determine the inhibitory activity of the new compound on the proliferation of the ovarian cancer cells after compounds treatment. As the results showed in Figure 4, we can see that compared with the control group, compound **1** could obviously reduce the proliferation of the ovarian cancer cells, suggesting the excellent application values of the new compound on ovarian cancer. However, the biological activity of compound **2** was much lower than compound **1**.

### Compound obviously inhibited the estrogen receptor relative expression on the ovarian cancer cells

**Figure 5.**
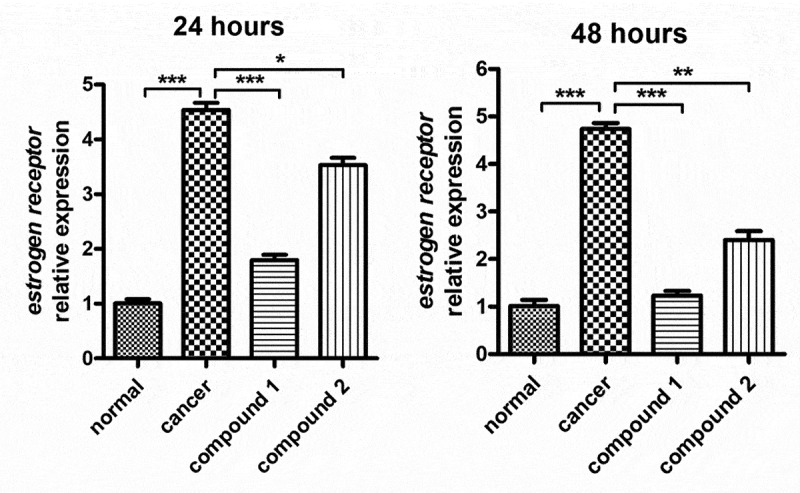
Obviously inhibited estrogen receptor relative expression on the ovarian cancer cells after compound treatment. The ovarian cancer cells in the logical growth phage were collected and seeded into the 6 well plates, the compound was added for treatment with indicated concentration. The relative expression levels of estrogen receptor on the ovarian cancer cells were determined with real time RT-PCR assay.

In the above experiment, we have proved that compound **1** could significantly reduce the proliferation of the ovarian cancer cells, which is much stronger than compound **2**. In addition to this, the estrogen receptor also plays an important role in the proliferation of ovarian cancer cells. So, the real-time RT-PCR assay was performed and the relative expression of the estrogen receptor on the ovarian cancer cells was determined. The results in Figure 5 showed that there was a significantly increased expression of estrogen receptor on the ovarian cancer cells compared with the control group. There was a significantly difference between these two groups, with P < 0.005. After the treatment of compound **1**, the estrogen receptor levels on the ovarian cancer cells were reduced, which is much stronger than the effect of compound **2**.

## Conclusion

In summary, we have successfully prepared two Cu(II) and Co(II)-containing coordination polymers via reaction of the corresponding metal salts with the H_4_L ligand. The as-prepared two coordination polymers have been studied via the single crystal X-ray diffraction, elemental analysis, powder X-ray diffraction and thermogravimetric analysis. The structural analysis results show that complex **1** features a 2D grid-like layer while complex **2** demonstrates a 4-connected 3D PtS framework based on 1D [Co(CO_2_)_2_]_n_ chains. The results of the CCK-8 assay showed that compound **1** could significantly reduce the proliferation of the new compound, which is stronger than compound **2**. In addition to this, the levels of the estrogen receptor on the ovarian cancer cells were also reduced significantly by compound **1**, but not compound **2**. In the end, we draw this conclusion, compound **1** could be an excellent candidate for ovarian cancer treatment by reducing the estrogen receptor on the ovarian cancer cells, which is stronger than compound **2**.
